# Understanding of facial features in face perception: insights from deep convolutional neural networks

**DOI:** 10.3389/fncom.2024.1209082

**Published:** 2024-04-09

**Authors:** Qianqian Zhang, Yueyi Zhang, Ning Liu, Xiaoyan Sun

**Affiliations:** ^1^MoE Key Laboratory of Brain-inspired Intelligent Perception and Cognition, University of Science and Technology of China, Hefei, China; ^2^Institute of Artificial Intelligence, Hefei Comprehensive National Science Center, Hefei, China; ^3^State Key Laboratory of Brain and Cognitive Science, Institute of Biophysics, Chinese Academy of Sciences, Beijing, China; ^4^College of Life Sciences, University of Chinese Academy of Sciences, Beijing, China

**Keywords:** face recognition, deep convolutional neural network, facial features, artificial face system, holistic processing

## Abstract

**Introduction:**

Face recognition has been a longstanding subject of interest in the fields of cognitive neuroscience and computer vision research. One key focus has been to understand the relative importance of different facial features in identifying individuals. Previous studies in humans have demonstrated the crucial role of eyebrows in face recognition, potentially even surpassing the importance of the eyes. However, eyebrows are not only vital for face recognition but also play a significant role in recognizing facial expressions and intentions, which might occur simultaneously and influence the face recognition process.

**Methods:**

To address these challenges, our current study aimed to leverage the power of deep convolutional neural networks (DCNNs), an artificial face recognition system, which can be specifically tailored for face recognition tasks. In this study, we investigated the relative importance of various facial features in face recognition by selectively blocking feature information from the input to the DCNN. Additionally, we conducted experiments in which we systematically blurred the information related to eyebrows to varying degrees.

**Results:**

Our findings aligned with previous human research, revealing that eyebrows are the most critical feature for face recognition, followed by eyes, mouth, and nose, in that order. The results demonstrated that the presence of eyebrows was more crucial than their specific high-frequency details, such as edges and textures, compared to other facial features, where the details also played a significant role. Furthermore, our results revealed that, unlike other facial features, the activation map indicated that the significance of eyebrows areas could not be readily adjusted to compensate for the absence of eyebrow information. This finding explains why masking eyebrows led to more significant deficits in face recognition performance. Additionally, we observed a synergistic relationship among facial features, providing evidence for holistic processing of faces within the DCNN.

**Discussion:**

Overall, our study sheds light on the underlying mechanisms of face recognition and underscores the potential of using DCNNs as valuable tools for further exploration in this field.

## 1 Introduction

In the field of face recognition research, the relative importance of each facial feature in achieving accurate face identification is a question of great interest (Davies et al., 1977). Previous studies have primarily focused on the eyes, mouth, and nose to determine the salience of these features and have established a hierarchy in which the eyes are deemed the most important, followed by the mouth and nose (Davies et al., 1977; Haig, 1986; Fraser et al., 1990). Surprisingly, subsequent studies have indicated that the eyebrows also play a crucial role in face recognition and maybe even more important than the eyes (Sadr et al., 2003). One potential explanation for the significance of eyebrows is their capacity to convey emotions and nonverbal signals (Sadr et al., 2003). That is, the importance of eyebrows in facial recognition may be confounded by their role in other facets of processing face-related information, such as the recognition of facial expressions (Maarten Milders and Logan, 2008) and judgments of personality (Bar et al., 2006; Willis and Todorov, 2006). Therefore, disentangling face recognition from other face-related information processing can enable a more precise assessment of the role of eyebrows in face recognition. However, achieving such disentanglement is challenging in human participants due to the concurrent and spontaneous processing of non-identity-related face information during perceptual face processing. Even when participants are explicitly instructed to perform face recognition tasks, the processing of non-identity-related face information may still occur and interact with the face recognition process. For example, personality traits such as trustworthiness and attractiveness are often rapidly and automatically inferred from facial appearance (Zebrowitz, 2004; Todorov et al., 2005; Wout and Sanfey, 2008; Antonakis and Dalgas, 2009).

Biologically inspired artificial neural networks, such as deep neural networks (DNNs), may offer an effective approach to addressing the abovementioned challenge. These networks have been designed to mimic the hierarchical architecture of the brain's visual processing pathway, consisting of feedforward projections with a linear-nonlinear neural motif (Cadieu et al., 2014; Yamins et al., 2014; Grossman et al., 2019). For example, Chakravarthi et al. proposed an automated CNN-LSTM model that incorporates the ResNet-152 algorithm (Chakravarthi et al., 2022). Compared to existing state-of-the-art methods, the newly proposed technique achieves an impressive accuracy rate of 98% by utilizing a hybrid deep learning algorithm, showcasing the effectiveness of DCNN in extracting facial features. As the same time, they exhibit similar characteristics in face processing to those in humans (Jacob et al., 2021; Tian et al., 2022). Notably, recent studies have shown that DCNNs pre-trained on facial datasets can replicate phenomena related to face processing, such as the Thatcher effect and face inversion effect (Jacob et al., 2021; Tian et al., 2022), suggesting that the realization process of the neural network is similar to that of face perception in the brain. Therefore, DCNNs have been utilized to access the underlying mechanisms of brain functions, such as the development of face perception (Baek et al., 2021; Jacob et al., 2021; Tian et al., 2022). Although DCNNs and the human brain may not operate on exact same principles, the comparable methodologies and existing literature demonstrate that DCNNs provide meaningful insights into the mechanisms of human face recognition. Notably, DCNNs can be purposefully trained to execute face recognition, thereby limiting the impact of other face-related information processing on the recognition process.

Recently, the mask method in Xie's study has demonstrated that certain features can be effectively suppressed in the network by setting the corresponding tensor values to zero (Xie et al., 2022), which means that the method successfully prevents any information related to the specific feature from flowing into the neural network system. This innovative approach offers valuable insights into studying specific features through the network. Using the mask method, researchers can gain a deeper understanding of how individual features influence the network's perception and recognition processes.

Here we employed ResNet (He et al., 2016), a DCNN known for its effective representation of face processing in the brain (Deng et al., 2019). ResNet has been proven to achieve state-of-the-art performance in face recognition (Meng et al., 2021; Kim et al., 2022). We selected the ResNet-101 as the backbone of our network to explore the relative importance of different facial features (i.e., eyebrows, eyes, mouth, and nose) and their combinations. First, we employed a feature detection-based network to detect the positions of each facial feature in face images. Next, instead of extracting these features from faces or replacing them with surrounding skin texture/color (Sadr et al., 2003), we implemented a masking technique to the feature region by assigning their values as zero. This approach allowed us to remove feature information while preserving the configural structure of faces. We fed these processed face images with missing feature information into the DCNN to explore the relative importance of facial features. Moreover, we varied the masking or blur levels on the facial features to examine whether recognition accuracy would decrease gradually as feature information was lost or suddenly at a specific blur level. Additionally, we employed the Gradient-weighted Class Activation Mapping (Grad–CAM; Selvaraju et al., 2017) to generate feature maps and explore the potential explanation of our findings.

## 2 Methods

### 2.1 Deep convolutional neural network

**Figure 1:**
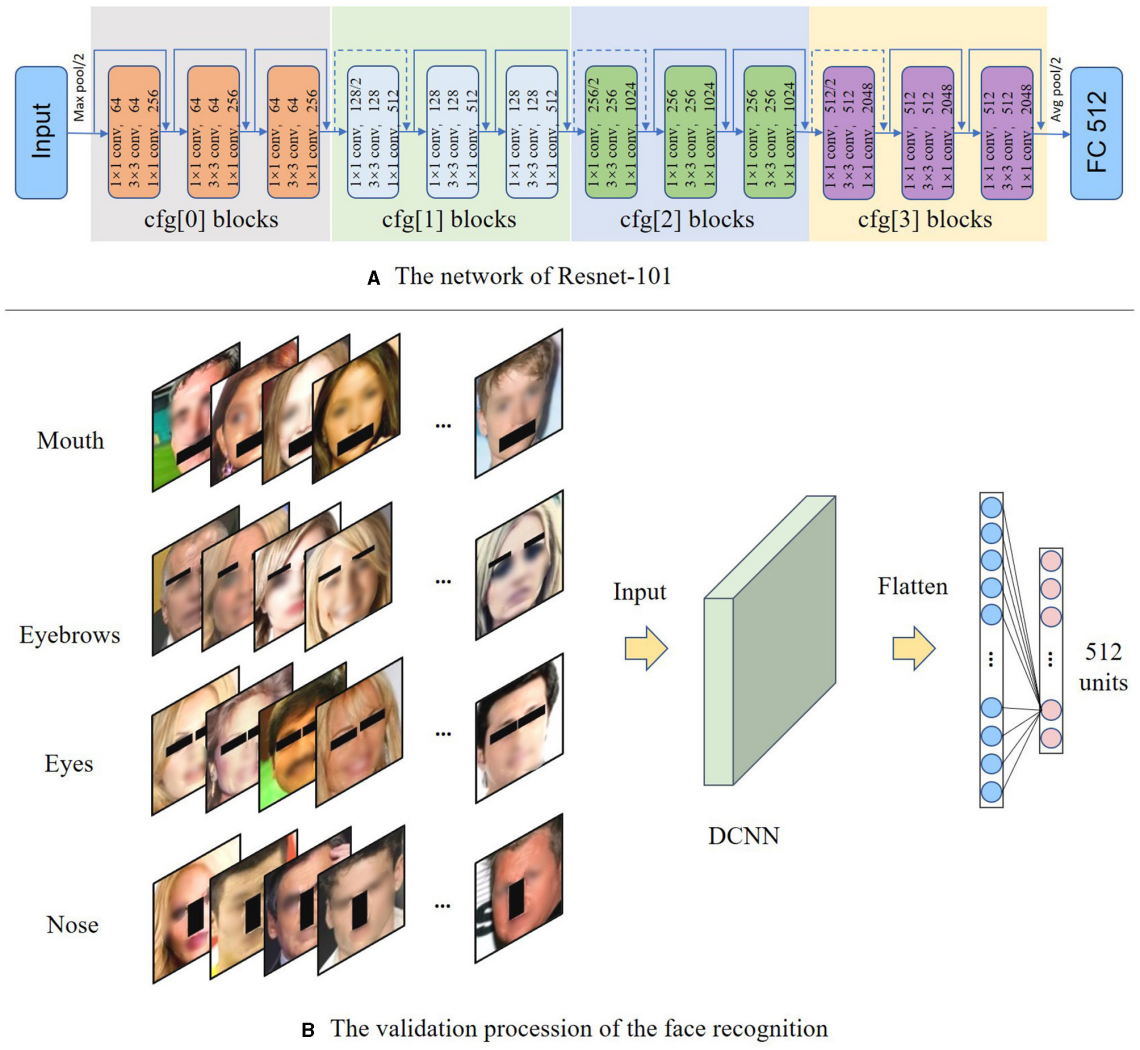
The validation procession of the face recognition. **(A)** The input of the network is a face image with a size of 112 × 112, and through the four blocks of ResNet. The final output is a 512-dimensional face classification result. **(B)** All the validation images are detected by the *S*^3^*FD* method (Zhang et al., 2017) The face features are then validated by DCNN using the validation dataset images corresponding to the masked features. To ensure privacy protection, the entire facial image was intentionally blurred.

ResNet (He et al., 2016) is a widely used DCNN backbone, which has been employed in various deep-learning tasks, including face recognition. For example, InsightFace achieves high performance in face recognition tasks by utilizing the ResNet as the backbone. Previous studies have demonstrated the effectiveness of ResNet as the backbone for obtaining a significant face representation that leads to better performance on downstream tasks, such as face classification or matching, compared to other convolutional networks (Meng et al., 2021; Kim et al., 2022). In the present study, we adopted the ResNet-101, which consisted of four blocks with varying convolution kernels, as the backbone to obtain face representation, as illustrated in Figure 1. To examine the impact of facial features on the accuracy of face recognition, we removed the last normalization layer and added a Fully Connected (FC) layer. Since our task was to classify faces into 512 classes, the final FC layer was a 512-unit classifier. We fed the network with face images of size 112 x 112, and obtained a classification result of 512 units. The overall data flow is shown in the following Algorithm 1.

**Algorithm 1:**
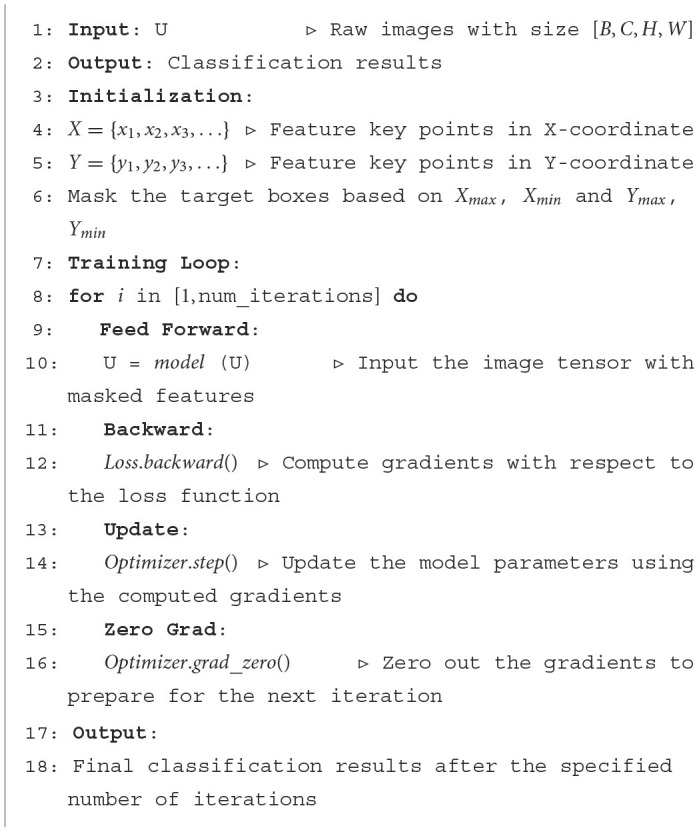
Face recognition algorithm.

For multi-classification tasks, there are *C* categories and the number of samples is *N*. The unnormalized score output by the model is *z*_*i, j*_, which represents the score of the *i*th sample on category *j*. The real label is *y*_*i, j*_, which represents the value (0 or 1) of category *j* in the real label of the *i*th sample. The cross-entropy loss is defined as Equation 1.


(1) $$\begin{array}{l} \text{CrossEntropyLoss}\left(y,z\right)=-\frac{1}{N}\overset{N}{\underset{i=1}{\sum }}\overset{C}{\underset{j=1}{\sum }}y_{i,j}\log\left(\frac{e^{z_{i,j}}}{\overset{C}{\underset{k=1}{\sum }}e^{z_{i,k}}}\right) \end{array}$$

where *N* represents the number of samples, and *C* represents the number of categories. This loss function measures the difference between the scores output by the model and the true labels. Through this loss function, we can constrain the model to focus solely on face classification tasks using the extracted features, thus avoiding interference from other tasks, such as emotion recognition and others. While the extracted feature information may still contain certain elements, such as emotional age, the constraints imposed by the loss function ensure that the semantic space remains aligned with the primary classification task.

Grad-CAM (Selvaraju et al., 2017) is a powerful visualization tool that illuminates the decision-making process of DCNN in face classification tasks. Grad-CAM introduced additional weights to the high-level features of the last layer, spatially correlating class-specific model activations with the input image. Specifically, Grad-CAM utilized the gradient information of the target class to weight each channel in the last layer's high-level features. By element-wise multiplication of these weighted feature maps with the output of the final convolutional layer, a heatmap was generated, indicating the correlation of each spatial location with the target class. By mapping the heatmap back to the original features, we generated an activation map, offering insights into the significance of various areas for the model's recognition process. This map illustrated the regions that contribute more significantly to the classification results in network decisions. The primary advantage of Grad-CAM lies in its non-intrusiveness, providing interpretability without requiring modifications to the network architecture. This facilitates a deeper comprehension of the correlation between the model's decisions and facial features, thereby amplifying insights into the decision-making process.

To enhance the reliability of our results, we conducted a comprehensive statistical analysis. Specifically, we performed bootstrap tests with a large number of iterations (*N* = 1,000). These tests simulated the distribution of recognition accuracy that would arise if the experiment were repeated with different sets of images.

### 2.2 Experiment settings

#### 2.2.1 The face datasets

In this study, we aimed to investigate the role of facial features in face recognition accuracy. To ensure the robustness and generalizability of our experimental results, we employed a rigorous dataset selection approach. Given the need for a broad spectrum of facial expressions and a substantial volume of training and validation data, we turned to extensive, high-quality datasets for support. Specifically, we chose three distinct datasets (face data1/2/3) from the extensive face dataset Face Emore (Guo et al., 2016; Deng et al., 2019). Each dataset comprised ~52,000 images, evenly distributed across 512 categories (identities), with equal representation of male and female subjects. To ensure fairness, we randomly sampled individuals from the Face Emore dataset, considering only those with at least 100 images to maintain balance and image quality. To conduct practical training and validation, we partitioned each dataset into a 7:3 ratio, with 36,400 images for training and 15,600 images for validation. Prior to training, to achieve better performance, we normalized the input images using the mean [0.485, 0.456, 0.406] and standard deviation [0.229, 0.224, 0.225] of the ImageNet datasets (Tian et al., 2022).

We employed the Single Shot Scale-invariant Face Detector (*S*^3^*FD*; Zhang et al., 2017) and a face alignment network (Bulat and Tzimiropoulos, 2017) to identify and locate the facial features, namely eyebrows, eyes, mouth, and nose. Regarding the mask method, we draw inspiration from the recent self-supervised reconstruction approach. (Xie et al., 2022) zero out the feature tensor to prevent relevant information from entering the network model, ensuring that the network disregards this specific portion of the feature information. During the masking process, facial feature keypoints were initially detected, followed by the connection of these keypoints to outline the respective feature areas. Subsequently, a rectangular region, precisely tailored to enclose all identified keypoints, was defined. This region, representing the smallest area capable of encompassing all keypoints, was then set to zero, effectively applying a mask to the designated feature. To ensure that the contribution of each feature to the network is not affected by other factors (e.g., size), we used a uniform masking area of 800 pixels, except for the eyebrows, which required a smaller area of 400 pixels due to their smaller size in Figure 1. To differentiate the effect of removing facial features from that of randomly obscuring parts of faces, we generated comparison data by randomly placing a mask of 800 or 400 pixels in each image. For each image, we generated random masks by selecting arbitrary regions based on the size and shape of the normal masked feature area. We utilized a random seed following a uniform distribution to ensure an even occurrence of random positions across all possible locations. As such, the locations of the random mask varied across images. Furthermore, in line with the real masks, we ensured that these random masks for different features did not overlap by employing a staged randomization process. Additionally, to maintain the integrity of the masking process within the image boundaries, we implemented a threshold at the image edges, thereby ensuring that the mask exclusively applies to the input image.

**Figure 2:**
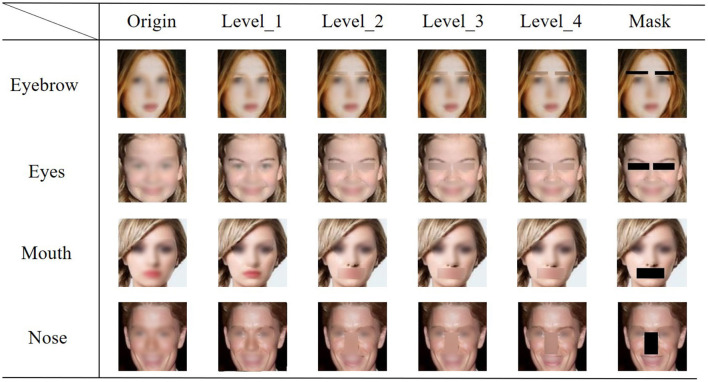
Visualization of the validation dataset with masks or various levels of blur. The horizontal axis represents the degree of blur, ranging from Level 1 to Level 4, with “Origin” indicating the original image. The vertical axis represents various facial features, such as eyebrows, eyes, mouth, and nose. To ensure privacy protection, the entire facial image was intentionally blurred.

Furthermore, to examine the influence of different degrees of blurring on recognition accuracy, we employed the Gaussian blur technique to mask the detected features to varying levels. Specifically, we applied the Gaussian blur method to create four levels of masks. Gaussian blur is a commonly used image processing technique, which gradually reduces feature information through Gaussian blur. Suppose the original image is *I*, and the Gaussian blurred image is *I*_blur_, the Gaussian blur operation can be expressed as the following convolution operation (Equation 2):


(2) $$\begin{array}{l} I_{\text{blur}}\left(x,y\right)=\overset{\frac{k}{2}}{\underset{i=-\frac{k}{2}}{\sum }}\overset{\frac{k}{2}}{\underset{j=-\frac{k}{2}}{\sum }}G\left(i,j\right)\cdot I\left(x+i,y+j\right) \end{array}$$

where *x* and *y* represent the pixel position of the image, *k* is the size of the Gaussian kernel (usually an odd number), and *G*(*i, j*) is the weight of the Gaussian kernel, which can be calculated by the following formula (Equation 3):


(3) $$\begin{array}{l} G\left(i,j\right)=\frac{1}{2\pi \sigma^{2}}\exp\left(-\frac{i^{2}+j^{2}}{2\sigma^{2}}\right) \end{array}$$

where σ is the standard deviation of the Gaussian kernel, which controls the degree of Gaussian blur. The value of σ directly correlated with the extent of blurring: a larger σ resulted in a more pronounced blur effect. To systematically analyze the impact of varying blur intensities, we incrementally adjusted σ from 2.0 to 20.0. This adjustment was designed to gradually increase the blurriness of the facial features. Specifically, we selected σ levels of 2, 8, 10, and 20, each corresponding to a distinct level of blurring, ranging from mild to severe. The highest level of blurriness was achieved in Mask, where pixels were set directly to zero, as shown in Figure 2. This fine-grained approach facilitated a comprehensive analysis of the contribution of individual facial features to the facial recognition process. Specifically, it allowed us to determine whether the recognition accuracy declined gradually with the incremental loss of feature details or if there was a threshold level at which the blurring effect became abruptly significant.

#### 2.2.2 Validation experiment

**Figure 3:**
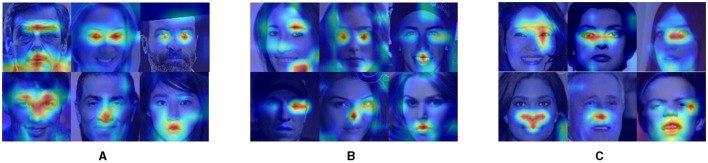
The heatmap of the validation procession on the three datasets. The red areas in these heatmaps represent features that the network emphasizes more during face recognition. **(A)** The heat map of original validation from the face_data_1. **(B)** The heat map of original validation from the face_data_2. **(C)** The heat map of original validation from the face_data_3.

The training process of the DCNN utilized intact face images without any feature masking (*n* = 36,400). Next, to evaluate the model's accuracy in face recognition, we conducted the validation with the remaining 15,600 intact face images. Concurrently, to ensure that the trained model can approach human-like face perception capabilities, we evaluated its performance on the Labeled Faces in the Wild (LFW) dataset (Huang et al., 2008), comparing it with human results (97.53%; Kumar et al., 2009). Then, images with masked facial features were employed to assess the model's sensitivity to facial features (Figure 3). The recognition accuracy of the DCNN was analyzed under various conditions, such as the masking of eyebrows, the masking of both eyebrows and mouth, and the different levels of masking applied to the eyebrows.

To further investigate the reliance of DCNN on facial features for face recognition, we employed the Grad-CAM (Selvaraju et al., 2017) to generate feature maps and then analyze the interpretability of DCNN. The Grad-CAM utilizes gradients of target units flowing into the final convolutional layer to produce a localization map highlighting the critical regions in the image for predicting the conception. We used the Grad-CAM to visualize the features on which DCNN counts for face recognition (Figure 3). To explore the degree of emphasis that DCNN devotes to each facial feature, we analyzed the weight of each feature in each tested image. The process was conducted in the following steps: inputting the dataset into the DCNN to obtain a heat map tensor for each image, matching the identified feature positions (mask position as described above) with the heat map tensor, calculating the weight of the corresponding area, and normalizing the weight based on the size of the region (800 pixels for eyes, nose, and mouth; 400 pixels for eyebrows) to determine the emphasis DCNN places on each feature.

To investigate the underlying factors contributing to fluctuations in facial recognition accuracy following the masking of specific facial features, we acquired feature maps from images in which various facial features were obscured and then conducted comparisons to access the changes in feature maps after masking certain facial features.

## 3 Results

### 3.1 Training results of the ResNet

In this study, we trained ResNet on three datasets to classify images of 512 face identities. To avoid overfitting, we terminated training after 30 epochs. The model yielded a validation accuracy exceeding 82% across all validation sets, significantly surpassing the 0.2% accuracy level obtained from the DCNN initialized with randomized weights (Table 1). In order to explore the impact of data augmentation, we used the masking data augmentation method as a comparison. The results were shown in the Supplementary Table 1. These results indicated that the ResNet model conducted in the present study exhibited reasonable and decent performance in face recognition.

**Table 1:** Performance comparison after masking single facial features.

**Dataset**	**Origin (%)**	**Random (%)**	**B (%)**	**E (%)**	**M (%)**	**N (%)**	**Importance Order**
Face_data_1	86.2	0.2	48.2^***^↓	33.1^***^↓	21.4^***^↓	13.7^***^↓	B>E >M >N ***
Face_data_2	87.1	0.2	43.7^***^↓	26.8^***^↓	17.9^***^↓	10.0^***^↓	B>E >M >N ***
Face_data_3	86.3	0.2	37.0^***^↓	30.4^***^↓	21.5^***^↓	19.8^***^↓	B>E >M >N ***
AVG	86.5	0.2	43.0^***^↓	30.1^***^↓	20.3^***^↓	14.5^***^↓	B>E >M >N ***

Origin represents the recognition accuracy on the intact face image validation set. Random indicates results with random weight initialization, serving as a control. B, E, M, and N represent the differences in recognition accuracy before and after masking eyebrows, eyes, mouth, and nose, respectively. The blue downward arrows indicate a decrease in recognition performance relative to the Origin after removing the corresponding facial feature. ^*^ denotes the significance of data compared to “Origin,” ^*^ denotes the significance of “Importance Order.” Statistical significance is denoted as follows: ^***^(p < 0.001).

To ensure that our model closely aligns with human face perception capabilities, we utilized the face encoder of our model, comprising all the ResNet models without the final fully connected (fc) layer. Subsequently, we evaluated its performance on the Labeled Faces in the Wild (LFW; Huang et al., 2008) public dataset for open-set face perception tasks. The accuracy achieved by the three models trained on our datasets on this open-set face perception task was 95.767, 94.164, and 94.958%, respectively. These results demonstrate a close resemblance to human recognition accuracy, as reported by LFW (97.53%; Kumar et al., 2009). Thus, after 30 epochs of training, our model exhibited face perception capabilities that were comparable to those of humans on the same dataset.

### 3.2 Recognition on face images with single facial feature masked

To investigate the contribution of each facial feature to face recognition, we first analyzed the impact of masking a single facial feature on the accuracy of face recognition. As shown in Table 1, the lowest recognition accuracy was obtained after masking eyebrows, followed by eyes, mouth, and nose. That is, eyebrows had the greatest impact on face recognition, consistent with the previous studies in humans. The findings demonstrated that the recognition accuracy after masking of features was significantly lower compared to the “Origin.” (*p* < 0.001), as shown in Table 1. This result underscored the critical importance of eyebrows in face recognition. While the masking data augmentation method was able to mitigate the influence of other facial features, it is important to note that eyebrows remained the most critical facial feature, even when employing this technique. Notably, despite the relatively smaller masked area for eyebrows compared to other features, their masking led to the most substantial impact on recognition accuracy. This outcome served to underscore the unique and critical importance of eyebrows within the context of facial feature contributions to accurate face recognition. In order to validate our findings that eyebrows are indeed the most crucial facial feature in DCNN, we replicated the experiment using alternative backbone architectures (i.e., VGG16 and ResNet-50) and different loss functions (i.e., cosface and arcface). The findings from these additional experiments align consistently with our earlier observations, further reinforcing the robustness and reliability of our conclusions. Details can be found in Supplementary Tables 2, 3.

To ensure that the decrease in face recognition accuracy is due to the absence of facial features rather than random variation, we conducted a comparative investigation using randomized masks, which were consistent in shape and size with those for facial features but were placed in random locations. The drop in the face recognition accuracy caused by randomized masks was 6.0% on average (6.6, 5.3, and 6.0% for the three datasets, respectively), which was significantly lower than the drop caused by masking the facial features (Table 1). These results indicated that facial features indeed played a more important role than facial non-feature parts in face recognition in the DCNN. Accordingly, the results substantiated that our conclusions stemmed not from random occurrences but from the specific contributions of the facial features.

To further investigate the impact of facial expressions on face recognition, we employed a two-step approach. Firstly, we utilized an expressions recognition network called MMNet (Li et al., 2022), which has been trained on the CASME2 dataset (Yan et al., 2014). The CASME2 dataset encompasses a variety of expressions such as happiness, disgust, surprise, and repression. This step enabled us to segment our validation set based on the identified expressions. Subsequently, we assessed the facial recognition performance on these segmented sub-datasets using our ResNet-100 model (refer to Supplementary Table 4). We observed similar face recognition accuracy across the different facial expression sub-datasets. Next, we investigated whether the significance of various facial features might vary across different facial expressions. To achieve this purpose, we once again applied masks to different facial features within each facial expression sub-dataset (refer to Supplementary Table 5). Our findings consistently revealed that the importance of eyebrows remained constant across the different facial expression sub-datasets.

### 3.3 Recognition results on face images with different degrees of blurring and masking on single facial features

To better understand the role of each facial feature in face recognition, we conducted a series of experiments, in we gradually increased the level of blurring applied to each feature from Level 1 to Level 4. We then evaluated the impact of masking and these four levels of blurring on the accuracy of face recognition.

**Figure 4:**
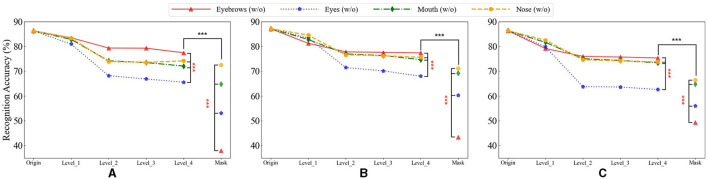
The recognition accuracy of different facial features of masks with different degrees on the three datasets. The horizontal axis represents varying levels of blurriness, ranging from mild blurring (Level 1) to severe blurring (Level 4), and Mask corresponds to a state where facial features are fully masked. Where Origin represents the result without mask. * denotes the significance of the results for “Eyebrows” in comparison with “Eyes,” “Mouth,” and “Nose.” ^*^ denote the significance of “Level_4” compared to “Mask” for each facial feature. Statistical significance is denoted as follows: ^***^(p < 0.001). **(A)** The results on the face_data_1. **(B)** The results on the face_data_2. **(C)** The results on the face_data_3.

As shown in Figure 4, as the level of blurring increased, the recognition accuracy of ResNet gradually decreased, reaching the lowest point when the features were fully masked. Notably, before Level 4, the decrease in recognition accuracy caused by blurring was the least significant on eyebrows among the four facial features. However, when eyebrows were fully masked, the recognition accuracy dropped significantly, much greater than the other three facial features. To verify these results, we performed a rigorous statistical significance analysis. The analysis confirmed a statistically significant difference (*p* < 0.001), indicating that the presence of eyebrows is crucial for face recognition but their detailed information may be less important.

In contrast, for the other three facial features, the influence of blur on face recognition spans from level 1 to level 4, including the mask condition. Notably, the relative importance ranking of these features remains consistent (Figure 4). These findings suggested that the detailed information of these features (especially eyes) might be important for face recognition.

### 3.4 Grad-CAM results of validation datasets masked single facial feature

To investigate the mechanisms underlying facial recognition within DCNNs, we employed the Grad- Cam visualization technique to assess the degree of emphasis of DCNNs to facial features. By employing heatmaps to visualize activation weights to the facial features (i.e., eyebrows, eyes, mouth, and nose) in three validation datasets, we found that DCNNs trained for facial recognition did focus on facial features and utilized either a singular feature or a combination of multiple features to achieve successful recognition (Figure 3).

Next, we sought to explore which features the DCNN emphasized more by calculating the normalized activation weight in each feature area (Table 2). Unexpectedly, our results showed that the degree of emphasis on eyes, noses, and mouth was similar to each other, with relatively low activation weights on eyebrows as compared to other features. These findings indicated that the most significant deficits resulting from masking eyebrows were not because DCNN emphasized this feature more. This finding aligned with outcomes from eye-tracking studies in human behavioral research (Iskra and Gabrijelčič, 2016; Jiang et al., 2019; Lim et al., 2020), which indicated that, compared to other facial features (like the eyes, nose, and mouth), the eyebrow area typically received less focus during face perception. This aligned with our observations that eyebrows received the least emphasis.

**Table 2:** The heatmap's activation weight (from 0 to 255) for the facial features.

**Features**	**Face_data_1**	**Face_data_2**	**Face_data_3**
Eyebrows	60.50	60.23	63.65
Eyes	70.51	68.47	68.84
Mouth	74.57	69.81	70.14
Nose	71.55	70.31	71.68

The weight value is calculated by averaging the activation weight of the feature area, which reflects the average activation weight of each facial feature by the DCNN.

**Figure 5:**
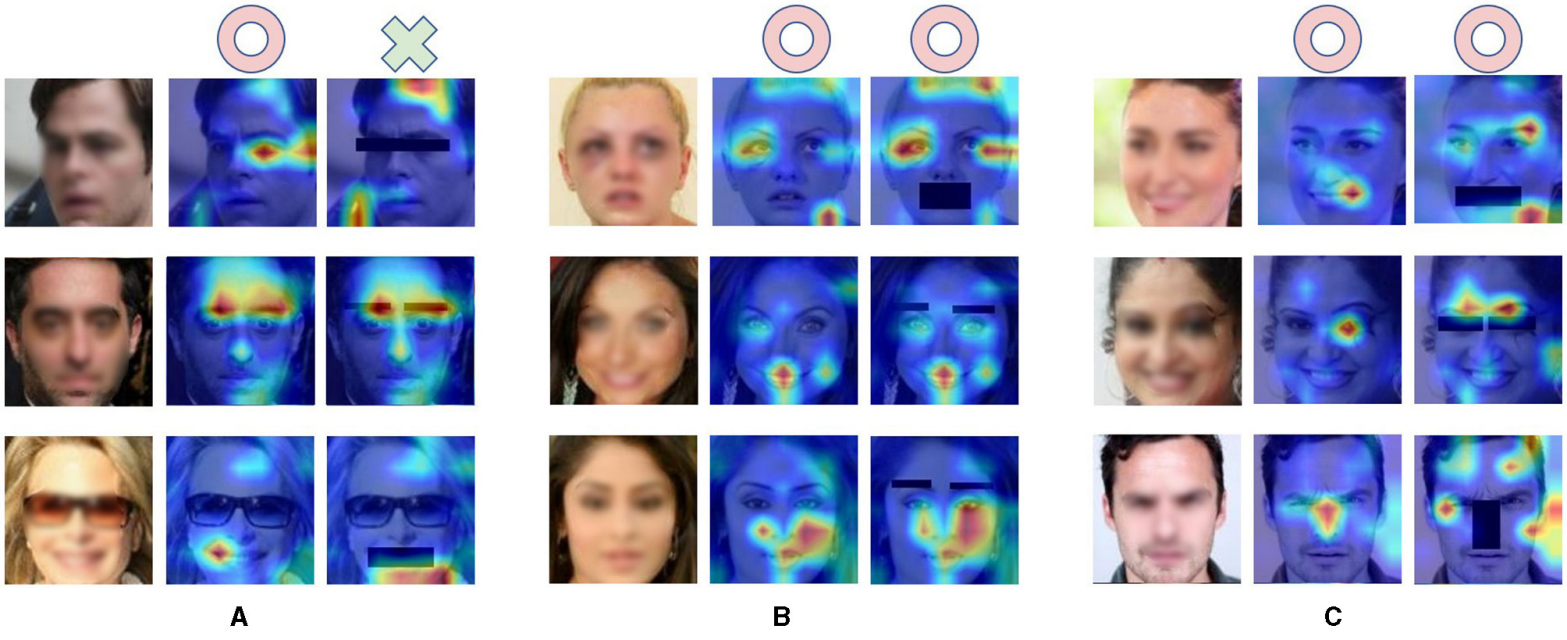
Visualization of Grad-CAM heatmaps illustrating the impact of masking facial features. **(A–C)** The first column shows the original image, while the second and third columns depict activation heat maps generated by the pre-trained network for the original and masked feature images. Red circles signify correct DCNN recognition, and green crosses indicate misidentification. To ensure privacy protection, the entire facial image was intentionally blurred.

To gain further insight into the deficits in face recognition caused by masking facial features, we compared the heatmaps for face images with and without masked facial features in Figure 5. We found that if the facial feature that the DCNN emphasized (e.g., eyes) was masked, the DCNN could not correctly recognize the face image, as illustrated in Figure 5A. Conversely, when less emphasized features were masked, the DCNN retained its proficiency in face recognition, as shown in Figure 5B. Notably, the DCNN could compensate for the absence of critical features by increasing its emphasis on alternative features, enabling correct face recognition as shown in Figure 5C. Thus, it is possible that the most significant deficits caused by masking eyebrows might be due to a lack of emphasis redirection. However, it is crucial to acknowledge that this interpretation is speculative and *post hoc* in nature.

**Figure 6:**
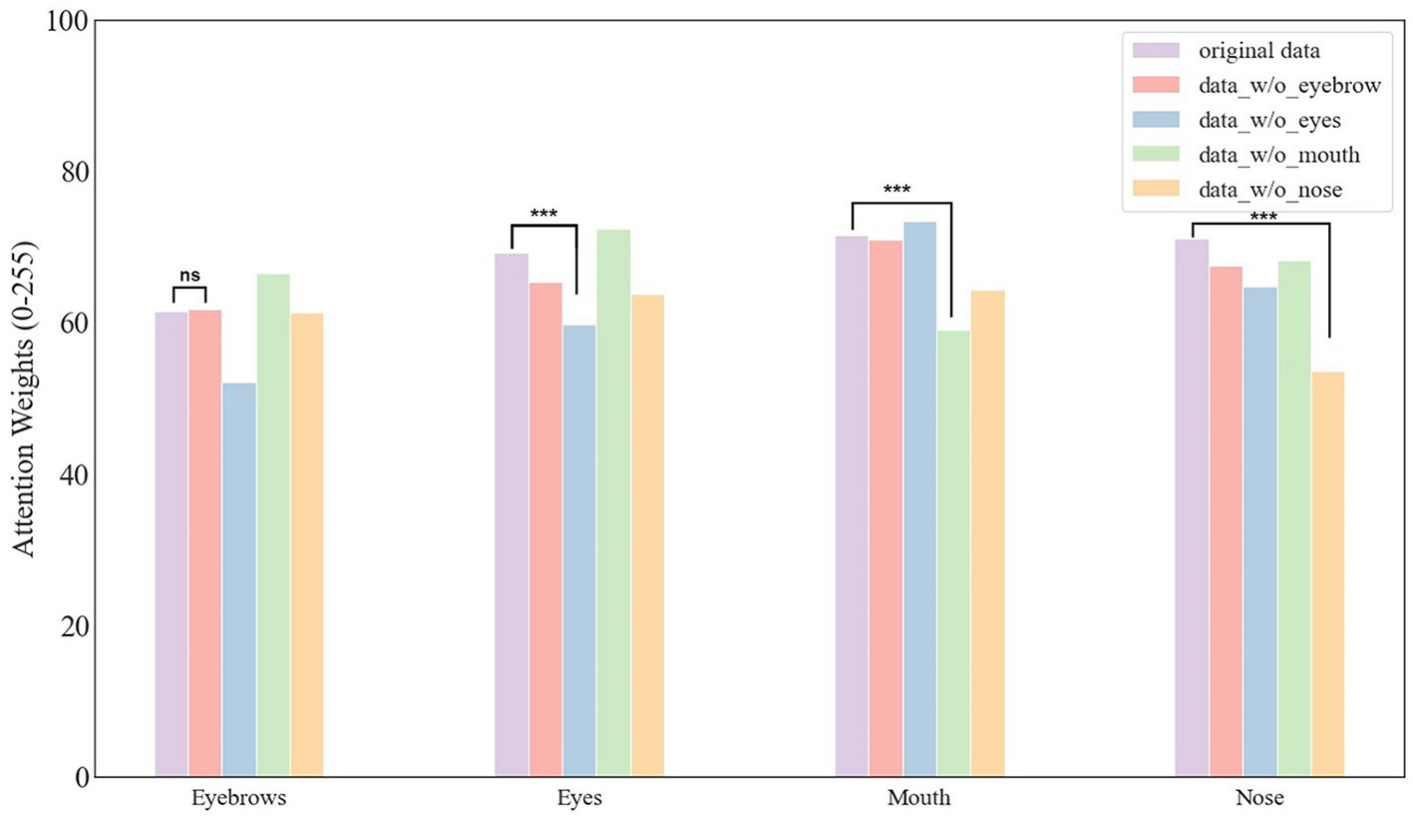
The activation weight of facial features. It is worth noting that, different from the case with eyebrows, the network paid the least emphasis to the feature that has been masked. Statistical significance is denoted as follows: ^***^(p < 0.001). “ns” denotes the there is no significant difference between the two bars.

To explore this possibility, we calculated the normalized activation weight for face images with masked features (Figure 6). It is important to note that the Grad-cam method normalized the activation map prior to allocating weights. Therefore, a reduction in activation for a masked feature implied that emphasis had been reallocated to other, unmasked features. We found similar results across the eyes, nose, and mouth, but not the eyebrows. Specifically, for example, when the eyes were masked, the corresponding region received less emphasis than when the eyes were intact, indicating that the DCNN shifted its emphasis to other facial features to compensate for the missing information. Conversely, when the eyebrows were masked, the emphasis on the corresponding region remained similar to when eyebrows were intact. We did statistical analysis for this conclusion, the analysis showed that the conclusion indicated a statistically significant difference (*p* < 0.001) except masking eyebrows. Therefore, this lack of compensatory emphasis may explain the most significant deficits in face recognition observed when the eyebrows are masked.

We introduced an analysis to quantify the degree of emphasis redirection, as outlined in Table 3. Specifically, we computed the changes in emphasis around each masked facial feature. Our results revealed that correctly recognized images exhibited a more pronounced reduction in emphasis on the masked feature after masking, indicating a redirection of emphasis toward other facial features that contributed to the accurate recognition of faces. Conversely, incorrectly recognized images tended to maintain emphasis around the masked features or demonstrated a less reduced emphasis, leading to recognition errors. Notably, our results indicated that masking eyebrows resulted in a relatively lower degree of emphasis redirection compared to other facial features. This underscored a unique aspect of eyebrow information in face recognition within DCNNs: the network's inability to reallocate emphasis to compensate for the lack of eyebrow cues, highlighting the critical role eyebrows play in the network's recognition process.

**Table 3:** Quantitative metric measuring the change in the spatial distribution of activation weight.

**True or false examples**	**Eyebrows**	**Eyes**	**Mouth**	**Nose**
True	−8.53	−10.78	−15.45	−20.34
False	2.31	0.54	−4.75	−8.60
All	0.26	−9.53	−12.45	−17.55

The values in the table represent the sample mean of the activation degree difference after masking the corresponding feature. Negative values indicate reduced activation, while positive values suggest increased activation.

### 3.5 Recognition results on face images with masking on multiple facial features

Previous studies in humans have confirmed that face recognition is a holistic process whereby faces are processed as wholes rather than as a collection of parts or features. Thus, it would be interesting to investigate whether the impact of combined features on face recognition in DCNN would be greater than the sum of each feature's individual impact. To address this question, next, we masked two facial features and examined the resulting changes in face recognition performance.

As shown in Table 4, masking both eyebrows and mouth yielded the greatest effect on the accuracy of face recognition, followed by the combination of masking both eyes and mouth, eyebrows and nose, eyebrows and eyes, eyes and nose, and finally, mouth and nose. As the same, we employed a bootstrap statistical method to validate the significance of our results. This result indicated an expectation that EM is significantly greater than EN, which in turn is significantly greater than MN, and so on down to BN being the least in the sequence (*p* < 0.001). Our findings indicated that the influence of combined masking of facial features on face recognition appears to correlate with the distance between the two masked features. In particular, the farther apart these two features are, the more pronounced the effect on face recognition. Another plausible hypothesis is that the eyebrows and the mouth collectively contribute to framing the overall shape of the face.

**Table 4:** Comparison of performance after masking the combined features, where B refers to the eyebrows, E refers to the eyes, M refers to the mouth, and N refers to the nose.

**Dataset**	**Origin (%)**	**BM (B+M) (%)**	**EM (E+M) (%)**	**BN (B+N) (%)**	**BE (B+E) (%)**	**EN (E+N) (%)**	**MN (M+N) (%)**	**Importance Order**
Face_data_1	86.2	73.9^***^(69.6)↓	68.2^***^(54.5)↓	60.9^***^(61.9)↓	53.8^***^(81.3)↓	47.8^***^(46.8)↓	35.3^***^(35.1)↓	BM>EM >BN >BE >EN >MN ***
Face_data_2	87.1	65.3^***^(61.6)↓	63.4^***^(44.7)↓	63.0^***^(53.7)↓	59.1^***^(70.5)↓	40.8^***^(36.8)↓	32.5^***^(27.9)↓	BM>EM >BN >BE >EN >MN ***
Face_data_3	86.3	67.6^***^(58.5)↓	64.5^***^(51.9)↓	57.8^***^(56.8)↓	50.6^***^(67.4)↓	47.1^***^(50.2)↓	35.0^***^(41.3)↓	BM>EM >BN >BE >EN >MN ***
AVG	86.5	68.9^***^(63.3)↓	63.0^***^(50.4)↓	60.5^***^(57.5)↓	54.5^***^(73.1)↓	45.2^***^(44.6)↓	34.3^***^(34.8)↓	BM>EM >BN >BE >EN >MN ***

For example, EM represents the differences in recognition accuracy before and after masking the eyes and mouth at the same time, and E+M represents the sum of the differences in recognition accuracy before and after masking the eyes and mouth separately. The blue downward arrows indicate a decrease in recognition performance relative to the Origin after removing the corresponding facial feature. ^*^ denotes the significance of data compared to “Origin,” ^*^ denotes the significance of “Importance Order.” Statistical significance is denoted as follows: ^***^(p < 0.001).

Next, to further investigate the impact of combined masking, we compared the impact of combined features and the sum of the impact of each feature within the combination. We found that the combined contribution of multiple features to face recognition exceeded the sum of individual contributions of the features within the combination. For example, when the eyes and mouth were individually masked, the face recognition accuracy rate decreased by 33.1 and 21.4%, respectively. However, when both features were masked simultaneously, the face recognition accuracy rate declined by 68.2%, which was greater than 54.5%. Our results indicated that the existence of holistic face processing in DCNN. However, we observed an exception for the combination of masking both eyebrows and eyes. To explain this phenomenon, we analyzed the face images that could not be recognized correctly and found that the unrecognized face images resulting from masking eyebrows and those from masking eyes exhibited considerable overlap. The number of coincidence error samples accounted for 79.1% of masking eyes and 60.0% of masking eyebrows respectively. This substantial overlap pointed to a commonality in the importance of eyebrows and eyes in the network's recognition process, offering a plausible explanation for why the combined masking of both features didn't demonstrate holistic face processing.

Finally, we examined the effect of complete information loss of facial features on face recognition accuracy when all the facial features were masked. We found a significant decrease in face recognition accuracy upon masking all facial features. Specifically, the accuracy on the three datasets dropped from 86.2, 87.1, and 86.3% when no features were masked to 7.9, 8.7, and 9.7% when all features were masked, respectively, resulting in an average decrease of 77.8%. These findings indicated that facial features might play a crucial role in face recognition in DCNN.

## 4 Discussion

In this study, we utilized ResNet, a popular DCNN, to investigate the significance of facial features in the accuracy of face recognition within DCNNs. Given the specific objective of our study, which aimed to gain insights from DCNN for understanding facial features in face perception in humans, our findings were mainly from faces with frontal views and minimal occlusions. We did not investigate the potential influence of pose and occlusions on the importance of facial features in this study. However, these factors could be explored in future research to enhance further our understanding of the roles of facial features in face perception. Our findings revealed the pivotal role of facial features in the face recognition process of DCNNs, with eyebrows emerging as the most critical, followed by eyes, mouth, and nose. Furthermore, our results indicated that, unlike other facial features, the mere presence of eyebrows played a crucial role in face recognition in DCNNs, with less emphasis on their detailed information such as shape or size. Employing the Grad-CAM visualization technique, we explored the effects of masking eyebrows and observed that the most significant deficits may be attributed to a lack of emphasis redirection. Overall, our research provided novel insights into understanding the intricacies of face recognition processing within DCNNs.

### 4.1 The most deficits in face recognition in DCNNs caused by masking eyebrows

In the present study, we delved into the relative importance of different facial features in DCNNs for face recognition by systematically masking them to eliminate their contribution. Our results unveiled that masking eyebrows led to the most pronounced deficits in face recognition, followed by masking eyes, mouth, and nose. These findings corroborated with previous studies conducted in humans. Earlier research had highlighted that eyes held the highest saliency among facial features, followed by the mouth and nose (Davies et al., 1977; Haig, 1986; Fraser et al., 1990). Previous investigations of face recognition had largely overlooked the role of eyebrows. However, when eyebrows were taken into account, they were found to be even more important than eyes. For example, the absence of eyebrows in familiar faces leads to the most substantial disruption in recognition performance (Sadr et al., 2003). Such a finding was surprising, given the common intuition that eyes are the most distinctive and expressive facial feature. The reasons for this phenomenon remain elusive. Several hypotheses have been proposed. First, eyebrows are essential in conveying emotions and other nonverbal signals. That is, eyebrows are important for facial expression and intention recognition, which may co-occur with face recognition automatically and thus affect it. Second, eyebrows' diversity of appearance across different faces may make them more attractive and informative for facial recognition (Sadr et al., 2003). Nevertheless, investigating these hypotheses in human subjects proves challenging, as disentangling the impact of eyebrows on emotion processing from face recognition involves intricate interactions with other face-related information processing.

In the present study, by taking advantage of DCNN, we explored the potential reasons for the importance of eyebrows in face recognition. We obtained similar results in three datasets, each consisting of ~52,000 images evenly distributed across 512 categories (i.e., identities), with equal representation of male and female subjects. Importantly, our findings suggested a dissociation between the level of activation and the importance of eyebrows in face recognition. In fact, eyebrows were the feature that received the least emphasis. Consequently, The importance of eyebrows was unlikely to be caused by more emphasis directed toward them. Moreover, the DCNN was constrained to focus only on the task of face recognition. That is, the emotional information and nonverbal signals conveyed by eyebrows cannot account for the findings related to their importance. Furthermore, the results of the blurring experiments showed that the effect of such blurring on the role of eyebrows in face recognition was very limited, as compared to other features. That is to say, even if there were variations in the appearance of eyebrows across different faces, at least the DCNN did not require the detailed information carried by eyebrows for accurate face recognition. Our Grad-CAM visualization technique further suggested that the notable deficits caused by masking eyebrows might be attributed to a lack of redirection in emphasis. In other words, even when the eyebrows are masked, DCNNs mistakenly maintain their focus on the eyebrows, while for other facial features, they adjust their emphasis to compensate for the missing information.

Additionally, we conducted blurring experiments to explore the distinct contributions of eyebrows and other facial features to face recognition in DCNNs. Notably, even at Level 4 of blurring, blurring eyebrows had lesser impact on face recognition compared to blurring other features. However, when the eyebrows were fully masked, the recognition accuracy dropped significantly, much greater than the other three facial features. Maximum blurring, while causing a loss of fine-grained information, retained average information corresponding to the blurred facial feature due to the blurring algorithm's principle, resulting in a smoother appearance for the blurred area. On the other hand, the full mask ensured that the neural network model remained entirely unaware of the masked features during the processing of other features. That is, for DCNNs, the rough information (e.g., location) of facial feature might still exist in the maximum blurred images but not in the fully masked images. Therefore, one possible explanation for our finding was that the position of eyebrows might facilitate the accurate analysis of facial information, which might explain their heightened importance in face recognition.

### 4.2 Holistic face processing in DCNNs

In human behavioral studies, various experimental paradigms have been extensively employed to investigate holistic face processing (Richler et al., 2012; Richler and Gauthier, 2014). Two notable examples are the composite face effect (Young et al., 1987; Murphy et al., 2017) and the part-whole effect (Tanaka and Farah, 1993; Tanaka and Simonyi, 2016). The composite face effect demonstrates that two identical top halves appear different when aligned with other bottom halves but not when the two halves are misaligned. In the part-whole task, the perception of a facial feature, such as the eyes, is more accurate when presented within an upright face than when shown in isolation. Consistent with the holistic view, recognition of the facial part is more accurate when tested in the whole-face condition than in isolation. Both the composite and part-whole effects suggest that facial features observed within a whole-face context are integrated rather than independently represented and processed.

In our current study, we did not manipulate the facial features as in the composite face or part-whole task. Although we did not extract features from the faces, we applied the masking techniques to compare the performance of DCNNs across different kinds of masking conditions. Specifically, we compared the performance of DCNNs on the images with single feature masked and those with combined feature masked. Although this represents an opposing operation, the underlying implication remains similar to those in the composite or part-whole effects. Notably, our findings revealed that the concurrent masking of two features exerted a more significant influence on face recognition compared to the combined effects of separately masking each feature. Despite the non-linear softmax activation function applied to classification outputs, it's essential to note that the final stages of classification involve a linear operation, particularly in the fully connected (fc) layer. The application of softmax is dedicated to probability computation, creating a 0–1 probability distribution. This step, which lacks trainable parameters and employs the already processed results from the fc layer for probability distribution construction, maintains the linearity of feature changes in the classification task. Consequently, if two features are independent, masking both should result in linear changes, combining the effects caused by each individual feature. The independence of features enables the network to model distinct feature vectors, leading to decoupled high-level semantic features tailored for the facial recognition task. If a non-linear change is observed in a combined mask, it suggests that the two features are not independent, potentially indicating the presence of holistic processing. Therefore, our findings indicated that DCNNs may process facial features in an integrated manner rather than independently, that is, DCNNs may engage in holistic processing when perceiving and recognizing faces, akin to the manner in which humans process faces, rather than treating facial features as isolated entities.

## 5 Conclusion

Taken together, our study examined the relative importance of specific facial features in face recognition using DCNNs. The findings revealed that eyebrows are the most critical feature for face recognition in DCNNs, which aligns with previous human-based studies. Our study overcame limitations encountered in human studies and confirms that the importance of eyebrows cannot be attributed to factors such as the diversity of eyebrow details or their conveying emotional and other nonverbal signals. Instead, our findings suggested that, different from other features, the presence of eyebrows, rather than their detailed information, is critical for the role of eyebrows in face recognition. Importantly, our results also indicated that, unlike other features, DCNNs cannot redirect emphasis to compensate for the absence of eyebrow information. Furthermore, our findings suggested that DCNN may also process faces in a holistic manner as humans.

Our study provided new insights into the neural mechanisms underlying face recognition and highlights the potential of using DCNNs as a tool to further explore this field. Future studies could investigate our findings on eyebrow processing in face recognition in humans.

## Data availability statement

Publicly available datasets were analyzed in this study. This data can be found at: https://github.com/SA21218172/Facial-Feature-Understanding-with-DCNNs.

## Author contributions

QZ: conceptualization, methodology, investigation, data curation, writing—original draft, and visualization. YZ: supervision and writing—review and editing. NL: methodology, formal analysis, writing—review and editing, and supervision. XS: writing—review and editing, supervision, and funding acquisition. All authors contributed to the article and approved the submitted version.
