# Supplementary material for: Understanding of facial features in face perception: insights from deep convolutional neural networks
Source: Front Comput Neurosci. 2024 Apr 9;18:1209082. doi: 10.3389/fncom.2024.1209082 (PMC11035738; doi:10.3389/fncom.2024.1209082)
Supplement: Supplementary file 1 [file Data_Sheet_1.PDF]

frontiers

Supplementary Material

1 SUPPLEMENTARY TABLES

1.1 Tables

Table S1. Validation performance of ResNet-100 on Masking Data Augmentation. "Val" represents the validation set, "Eyebrows" denotes the validation set after masking eyebrows, "Eyes" after masking eyes, "Mouth" after masking mouth, and "Nose" after masking nose. The values in parentheses denote the recognition accuracy without data augmentation.

	Val	Eyebrows	Eyes	Mouth	Nose
Face_data1	86.6% (86.2%)	66.3% (38.0%)	67.3% (53.1%)	76.2% (64.8%)	76.5% (72.5%)
Face_data2	86.4% (87.1%)	63.1% (43.4%)	70.4% (60.3%)	74.5% (69.2%)	75.0% (71.1%)
Face_data3	85.7% (86.3%)	65.3% (49.3%)	68.5% (55.9%)	72.8% (64.8%)	74.1% (66.5%)

Table S2. Performance comparison of three models on different datasets. "Val" represents the validation set, "Eyebrows" denotes the validation set after masking eyebrows, "Eyes" after masking eyes, "Mouth" after masking mouth, and "Nose" after masking nose.

(a) Validation performance of VGG-16

	Val	Eyebrows	Eyes	Mouth	Nose
Face_data1	82.3%	23.1%	31.0%	51.6%	39.0%
Face_data2	81.6%	9.9%	16.7%	43.3%	27.2%
Face_data3	86.9%	27.3%	33.4%	55.6%	44.6%

(b) Validation performance of VGG-19

	Val	Eyebrows	Eyes	Mouth	Nose
Face_data1	85.0%	24.9%	25.2%	46.2%	31.7%
Face_data2	83.9%	17.3%	19.9%	42.0%	28.2%
Face_data3	84.0%	24.6%	24.6%	38.1%	29.1%

(c) Validation performance of Resnet-50

	Val	Eyebrows	Eyes	Mouth	Nose
Face_data1	87.4%	31.8%	49.3%	60.6%	68.2%
Face_data2	85.0%	42.1%	54.7%	60.1%	67.2%
Face_data3	88.0%	45.7%	54.7%	67.3%	70.1%

Table S3. Validation performance of ResNet-100 on different Loss Functions. ”Val” represents the validation set, ”Eyebrows” denotes the validation set after masking eyebrows, ”Eyes” after masking eyes, ”Mouth” after masking mouth, and ”Nose” after masking nose. The results represent recognition accuracy, with values in parentheses indicating recognition accuracy using Cross Entropy as the Loss Function.

(a) Validation performance on arcface Loss.

	**Val**	**Eyebrows**	**Eyes**	**Mouth**	**Nose**
Face_data1	90.4% (86.2%)	33.8% (38.0%)	49.9% (53.1%)	70.5% (64.8%)	66.4% (72.5%)
Face_data2	89.7% (87.1%)	39.0% (43.4%)	52.7% (60.3%)	73.3% (69.2%)	68.1% (71.1%)
Face_data3	90.9% (86.3%)	38.1% (49.3%)	55.3% (55.9%)	70.9% (64.8%)	71.3% (66.5%)

(b) Validation performance on cosface Loss.

	**Val**	**Eyebrows**	**Eyes**	**Mouth**	**Nose**
Face_data1	91.5% (86.2%)	39.3% (38.0%)	57.3% (53.1%)	71.9% (64.8%)	74.0% (72.5%)
Face_data2	89.2% (87.1%)	35.1% (43.4%)	46.4% (60.3%)	70.7% (69.2%)	71.1% (71.1%)
Face_data3	90.8% (86.3%)	31.3% (49.3%)	44.4% (55.9%)	66.6% (64.8%)	69.6% (66.5%)

Table S4. Validation performance of ResNet-100 on Various Expressions and Overall Val.

	**Happiness**	**Disgust**	**Surprise**	**Repression**	**Other**	**Val**
Face_data1	86.3%	85.8%	86.1%	85.8%	86.1%	86.2%
Face_data2	87.0%	87.5%	86.6%	86.7%	87.2%	87.1%
Face_data3	86.6%	86.4%	86.7%	86.1%	85.9%	86.3%

Table S5. Validation performance of ResNet-100 with facial features masking on various expressions and overall validation set. ”Val” and expressions such as “Happiness” represent the recognition accuracy after masking specific facial features.

(a) Recognition accuracy on masking eyebrows across various expressions.

	**Happiness**	**Disgust**	**Surprise**	**Repression**	**Other**	**Val**
Face_data1	38.3%	38.2%	38.0%	38.0%	37.7%	38.0%
Face_data2	42.5%	43.1%	43.6%	42.9%	43.0%	43.4%
Face_data3	48.9%	50.6%	49.2%	49.3%	49.4%	49.3%

(b) Recognition accuracy on masking eyes across various expressions.

	**Happiness**	**Disgust**	**Surprise**	**Repression**	**Other**	**Val**
Face_data1	53.3%	53.0%	52.8%	53.2%	52.6%	53.1%
Face_data2	58.9%	60.9%	58.8%	61.5%	60.6%	60.3%
Face_data3	56.9%	56.5%	55.3%	55.5%	55.6%	55.9%

(c) Recognition accuracy on masking mouth across various expressions.

	**Happiness**	**Disgust**	**Surprise**	**Repression**	**Other**	**Val**
Face_data1	65.7%	64.3%	64.5%	65.3%	64.4%	64.8%
Face_data2	69.9%	70.0%	68.6%	68.8%	69.1%	69.2%
Face_data3	64.5%	66.1%	64.1%	63.8%	64.4%	64.8%

(d) Recognition accuracy on masking nose across various expressions.

	**Happiness**	**Disgust**	**Surprise**	**Repression**	**Other**	**Val**
Face_data1	73.6%	72.8%	72.0%	71.8%	72.2%	72.5%
Face_data2	71.5%	72.2%	70.7%	71.1%	70.9%	71.1%
Face_data3	66.4%	66.6%	67.2%	65.9%	65.7%	66.5%
